# Bed-Sharing in Couples Is Associated With Increased and Stabilized REM Sleep and Sleep-Stage Synchronization

**DOI:** 10.3389/fpsyt.2020.00583

**Published:** 2020-06-25

**Authors:** Henning Johannes Drews, Sebastian Wallot, Philip Brysch, Hannah Berger-Johannsen, Sara Lena Weinhold, Panagiotis Mitkidis, Paul Christian Baier, Julia Lechinger, Andreas Roepstorff, Robert Göder

**Affiliations:** ^1^Department of Psychiatry and Psychotherapy, Christian-Albrechts University Kiel, Kiel, Germany; ^2^Department of Language and Literature, Max Planck Institute for Empirical Aesthetics, Frankfurt am Main, Germany; ^3^Department of Psychology, Christian-Albrechts University Kiel, Kiel, Germany; ^4^Department of Management, Aarhus University, Aarhus, Denmark; ^5^Center for Advanced Hindsight, Social Science Research Institute, Duke University, Durham, NC, United States; ^6^Interacting Minds Centre, Aarhus University, Aarhus, Denmark

**Keywords:** co-sleep, REM sleep, synchronization, bed-sharing, physiological coupling, sociality, chronotype, relationship quality

## Abstract

**Background/Objectives:**

Sharing the bed with a partner is common among adults and impacts sleep quality with potential implications for mental health. However, hitherto findings are contradictory and particularly polysomnographic data on co-sleeping couples are extremely rare. The present study aimed to investigate the effects of a bed partner's presence on individual and dyadic sleep neurophysiology.

**Methods:**

Young healthy heterosexual couples underwent sleep-lab-based polysomnography of two sleeping arrangements: individual sleep and co-sleep. Individual and dyadic sleep parameters (i.e., synchronization of sleep stages) were collected. The latter were assessed using cross-recurrence quantification analysis. Additionally, subjective sleep quality, relationship characteristics, and chronotype were monitored. Data were analyzed comparing co-sleep vs. individual sleep. Interaction effects of the sleeping arrangement with gender, chronotype, or relationship characteristics were moreover tested.

**Results:**

As compared to sleeping individually, co-sleeping was associated with about 10% more REM sleep, less fragmented REM sleep (*p* = 0.008), longer undisturbed REM fragments (*p* = 0.0006), and more limb movements (*p* = 0.007). None of the other sleep stages was significantly altered. Social support interacted with sleeping arrangement in a way that individuals with suboptimal social support showed the biggest impact of the sleeping arrangement on REM sleep. Sleep architectures were more synchronized between partners during co-sleep (*p* = 0.005) even if wake phases were excluded (*p* = 0.022). Moreover, sleep architectures are significantly coupled across a lag of ± 5min. Depth of relationship represented an additional significant main effect regarding synchronization, reflecting a positive association between the two. Neither REM sleep nor synchronization was influenced by gender, chronotype, or other relationship characteristics.

**Conclusion:**

Depending on the sleeping arrangement, couple's sleep architecture and synchronization show alterations that are modified by relationship characteristics. We discuss that these alterations could be part of a self-enhancing feedback loop of REM sleep and sociality and a mechanism through which sociality prevents mental illness.

## Introduction

Romantic relationships influence mental health (1). Sleep has been argued to mediate this relationship (2). In this context, sharing a bed with a partner is of special interest since it expands the relational interaction into the night. However, the actual effects of bed sharing on objective sleep measures are an open question, since hitherto findings are diverse:

Actigraphic studies of human couples comparing co-sleep to individual sleep report co-sleep to be either linked to more disrupted sleep patterns in both sexes (3) or in women only (4) or to be linked to increased sleep time in men (5). Actigraphic between subjects comparisons show longer total sleep time (TST), and less time awake after sleep onset for married couples compared to unmarried single controls (6). Furthermore, synchronization of movements (3) and increased sleep wake concordance during co-sleep (7) have been reported. Both, individual and dyadic parameters, seem to be influenced by relationship characteristics such as partner conflict or marital quality (7, 8).

However, actigraphy calculates sleep from body movements and does not allow for neurophysiological assessment (i.e., monitoring of sleep stages). This is an important restriction since many beneficial effects of sleep, e.g., memory formation, social functioning, or mental health effects, have been directly linked to certain sleep stages and specifically to slow-wave sleep (SWS) and REM sleep (9–16). So far, only two polysomnographic studies exist that compare co-sleeping and individual sleep of healthy couples (17, 18) and one of these studies is a small pilot study of the present work (17). Interestingly, while both report an increase of REM sleep during co-sleep other findings (regarding SWS, sleep latencies, TST, sleep efficiency, awakenings, and subjective sleep parameters) differ between the studies. This heterogeneity renders the current picture of the neurophysiology of social sleep inconclusive, and it is a standing question whether co-sleeping couples sleep better, worse, or just different.

Moreover, additional (potentially) relevant phenomena have only been insufficiently addressed in the above-mentioned polysomnographic studies: Neither study has included relationship characteristics or chronotype as covariates, and only our pilot study has looked at direct synchronization of sleep stages (17), missing out more complex forms of coupling (e.g., lead and lag phenomena) as well as the relevance of relationship characteristics and chronotype for sleep stage synchronization. However, addressing sleep-stage synchrony during sleep might be particularly interesting since interpersonal synchronization during wakefulness has been related to prosocial behavior, perceived social bonding, social cognition, and positive affect [for review see (19)] - all of which are important in the context of mental illness.

Therefore, we investigated the effect of the presence of the partner on young healthy couples' sleep by use of sleep-lab-based dual simultaneous polysomnography, and cross-recurrence quantification analysis (20).

Conceptually, the study comprises two aspects. First, a confirmatory part that re-assesses the results of the pilot study in a bigger sample and assesses the effects of a bed partner on objective sleep parameters and direct sleep-stage synchrony. Second, an exploratory part that investigates i) the relevance of relational and individual factors (e.g., relationship quality, gender, chronotype) for the changes in sleep outcomes and synchrony and ii) more complex forms of interpersonal coupling such as lead-and-lag phenomena (i.e., intra-couple synchronization that occurs with a certain time delay). The first aspect seeks to answer the question whether couples sleep better, worse, or just different, the second aspect further explores the understudied field of bed sharing in adult couples.

## Materials and Methods

### Sample

For the present study, we recruited 24 childless healthy young adults (target age group: 18 to 29 years), belonging to 12 heterosexual couples with a history of co-sleeping with the same partner on the majority of nights per week for at least 3 months prior to study initiation. Inclusion criteria were absence of shift work, pregnancy, and medications or disorders known to affect sleep (including depression, addictions, and sleep disorders). Compliance with inclusion criteria was assessed by a clinical interview. Additionally, inconspicuous results in the Beck's Depression Inventory (21), the Alcohol Use Disorders Identification Test (AUDIT) (22), the revised Cannabis Use Disorders Identification Test (CUDIT-R) (23), the Pittsburgh Sleep Quality Index (PSQI) (24), and the Epworth Sleepiness Scale (ESS) (25) were required for study inclusion (see **Table 1** for sample characteristics including results of the above inventories).

**Table 1:** Sample and relationship characteristics.

(n = 24)	Mean	SD(±)
Age [years]	23.5	3.0
Scholarly education [years]	12.9	2.0
Relationship duration [months]	34.0	28.0
Quality of Relationship Inventory support	3.7	0.3
Quality of Relationship Inventory depth	3.5	0.3
Quality of Relationship Inventory conflict	1.5	0.3
Hatfield Passionate Love Scale	85.2	8.9
Bed-sharing [months]	19.1	11.7
Bed-sharing [days per week]	6.4	1.1
Pittsburgh Sleep Quality Index	2.9	1.3
Epworth Sleepiness Scale	4.7	3.1
Morningness–Eveningness Questionnaire	54.8	7.8
Beck's Depression Inventory	1.9	2.3
Alcohol Use Disorders Identification Test	3.7	3.0
Cannabis Use Disorders Identification Test	0.3	1.3

### Procedure

Prior to study initiation, ethical clearance by the ethical board of Kiel University's Medical Faculty and written informed consent was obtained. To control for the large interindividual differences in sleep architecture (26) and to obtain a significant statistical power with a moderate sample size, a within subjects design was chosen. Couples spent four nights on two consecutive weekends in the sleep laboratory undergoing individual and dual, simultaneous polysomnography. Sleeping arrangement (sleep with a partner or individual sleep) within one weekend was kept constant but was altered between weekends so that every couple slept individually on one weekend and with a partner on the other weekend. The order of sleeping arrangements was counterbalanced across all couples, with half of the couples starting with individual sleep and the other half with co-sleep. Individual sleep took place in single beds in separate rooms, co-sleep in single beds that were adjacent to each other. Two sheets and duvets were used; the cleft between the beds was bolstered so that a homogenous reclining area was guaranteed. The first night of each set was an adaptational night to the setting and the sleeping arrangement and was not included in the analysis. Also, the first night served to detect and possibly exclude people with sleep apnea or periodic limb movement disorder (none excluded).

Before and after every night, participants completed an evening and morning protocol. Questionnaires assessing the inclusion criteria and chronotype were completed before the first night. Measures of relationship quality were assessed before Night 2 and 4 (the mean of both was used for further analysis) and the Hatfield passionate love scale after night 4. To ensure a maximum of overlap in pre-sleep waking activity, couples assigned for individual sleep were separated just before going to bed.

### Measures

#### Objective Sleep Data – Polysomnography

Participants underwent full cardiorespiratory polysomnography monitoring EOG, EEG (F3, F4, C3, C4, O1, O2), chin-EMG, ECG, pulseoxymetry, EMG of both anterior tibial muscles, and respiratory parameters as flow and movements of chest and abdomen. Sleep stages were manually coded by one experienced, blinded rater according to the AASM criteria (27). Leg movements were calculated automatically, by the polysomnographs' standard software (Somnomedics Domino). A REM sleep period was defined as REM sleep belonging to one sleep cycle. REM sleep fragmentation was defined as any interruption (i.e., one or more epochs not scored as REM sleep) between two epochs of REM sleep of one sleep cycle. Average duration of interruption-free REM sleep fragments was calculated by dividing REM sleep duration by number of fragments.

Coupling of sleep stages between partners was determined using cross-recurrence quantification analysis as described by Marwan and colleagues (20). Cross-recurrence quantification analysis is a powerful statistical tool that is able to assess different layers of coupling (e.g., complete synchronization, phase synchronization, lag synchronization, or generalized synchronization) and is therefore highly useful for studying coupling of complex dynamic systems (20). It has been used in such diverse fields as neuroscience, economics, geophysics, and engineering (20). Furthermore, it has been introduced to the study of physiological coupling of co-sleeping couples in the pilot to the present work (17). Technically, cross-recurrence quantification analysis is a nonlinear correlation analysis for bi-variate time-series data. Its core tool is the cross-recurrence plot, which is a two-dimensional binary matrix where cross-recurrence between two time-series are charted. Here, a cross-recurrence is an instance where the two time-series take the same – or similar – values at a certain lag. Based on the cross-recurrence plot, several recurrence measures can be computed that quantify (nonlinear) correlation patterns between two time-series. Moreover, leader-follower relationships between two time-series can be computed based on cross-recurrence plot. That means that not only direct synchronization can be assessed (i.e., whether both time series are in the same state at the same time point) but also other forms of synchronization such as lag synchronization. Here the two time series are synchronized only if a certain time delay is considered (20). See Wallot and Leonardi (28) for an introduction to cross-recurrence plots and the quantification of leader-follower relationships.

#### Subjective Sleep Data

Subjective sleep onset latency, subjective sleep time, and subjective number of awakenings were assessed each morning immediately after waking up. Moreover, to cover subjective morning condition, we derived three sexpartite Likert subscales of morning condition (from feeling depressed (1) to lighthearted (6), run down (1) to refreshed (6), or tense (1) to relaxed (6)) from the morning and evening protocol of the German Sleep Medicine Society (DGSM) (29). The results of the scales were merged into a single morning-condition sum score. The chronotype was determined by use of the German version of the morningness–eveningness questionnaire (D-MEQ) (30). Here, higher ratings indicate an earlier chronotype. On the basis of the D-MEQ scores, subjects can be categorized into the following categories: definitely morning type (score, 70–86), moderately morning type (score, 59–69), neither type (score, 42–58), moderately evening type (score, 31–41), and definitely evening type (score, 16–30) (31).

#### Relationship Characteristics

Regarding relationship characteristics, we collected relationship duration, degree of passionate love, conflict, social support, and relationship depth. The latter three dimensions are part of the quality of relationship inventory (QRI) of which we use the German version (32). The QRI is a 25-item inventory in which a tetrapartite Likert scale (1= not true - 4 = almost always true) is used to answer questions like “How angry does this person make you feel?” (conflict), “To what extent could you count on this person for help with a problem?” (support), or “How significant is this relationship in your life?” (depth). In their validation study for the German version of the QRI, Reiner et al. report the following mean (± SD) values for the youngest age group (18–44 years; n = 508): 3.23 ± 0.57 (support dimension), 1.87 ± 0.52 (conflict dimension), and 3.25 ± 0.55 (relationship-depth dimension). It is of note that the QRI is not limited to romantic relationships and has been used to assess a variety of social relationships (e.g., mentoring-relationships (33), same-sex friends (34), or parents and children (35)). In order to additionally include a relationship dimension specific to romantic relationships, we assessed passionate love *via* the Hatfield passionate love scale, a 15-item scale with a septpartite Likert scale (1= not true at all – 7= absolutely true) (36). Exemplary statements are: “I want [name] physically, emotionally, mentally.” or “Sometimes I feel I can't control my thoughts; they are obsessively on [name].”

### Statistical Analysis

#### Analytical Procedure

To ensure comparability with the previous two studies that polysomnographically investigated co-sleeping vs. individually sleeping in healthy couples (17, 18), we aligned our statistical approach with these works.

First, we tested the relevance of sleeping arrangement (co-sleep vs. individual) for subjective and polysomnographic sleep outcomes (confirmatory part of the study). Dependent variables were subjective morning condition, subjective sleep onset latency, subjective total sleep time, subjective number of awakenings, polysomnographic total sleep time, polysomnographic sleep efficiency, polysomnographic sleep onset latency, polysomnographic REM sleep latency, polysomnographic amount of sleep stages N1, N2, N3, and REM sleep relative to total sleep time (% of total sleep time), polysomnographic number of awakenings, and isolated leg movements. Tests employed were paired, two-tailed Student's t-tests or – where applicable – the nonparametric alternative Wilcoxon signed-rang tests (WSR). Normal distribution was tested by the Shapiro-Wilk test. Alpha-inflation was countered by using the method of Benjamini & Hochberg, which is based on controlling the false discovery rate (37). Synchronization coefficients for lag 0 were compared using paired two-tailed Student's t-tests (co-sleep vs. individual sleep).

For the exploratory part of the study, we investigated lead and lag phenomena in coupling and the influence of additional factors (relationship characteristics, gender, chronotype, snoring, movements) on the significant parameters of part 1.

Yet, before exploring the effects of additional parameters, we first assessed the degree of dependence of individuals of each couple. Therefore, we correlated the couples' individuals with each other (males–females, Pearson correlations) as suggested by Kashy and Snyder (38). This was done to see whether an analysis on the couples' level (a dyadic approach) was necessary or an analysis on the individual level was justifiable. The results supported the analysis on the individuals' level and moreover—since this was the approach chosen by Monroe (18) to assess gender effects - ensured better comparability with this highly relevant study.

Thus, we conducted two-way mixed analyses of variance (ANOVAs) for the within factor SLEEPING ARRANGEMENT (co-sleep vs. individual sleep) and the between factor GENDER (male vs. female), as were analyses of covariance (ANCOVAs) for the independent within-variable SLEEPING ARRANGEMENT and the covariates CONFLICT, DEPTH of RELATIONSHIP, SOCIAL SUPPORT, PASSIONATE LOVE, and RELATIONSHIP DURATION, respectively. The ANCOVAs were also calculated with amount of sleep stage synchronization as dependent variable. Here CHRONOTYPE, SNORING, and LEG MOVEMENTS were inserted as additional covariates.

Significance across lags was defined by non-overlapping confidence intervals in the synchronization plots across lags.

#### Statistical Power and Sample Size Calculation

For the confirmatory aspect of the study, the sample size was calculated using a two-sided paired t-test (significance level 0.05) with a power of 0.8 based on a medium expected effect size of d = 0.6. The results of the pilot to the present work (17) have shown similar or larger effect sizes for subjective morning condition, subjective TST, sleep efficiency, total REM sleep, and REM-sleep percentage. This result leads to a required sample size of 24 subjects.

All analyses were calculated using R (Version 3.6.1) (39) and MATLAB [Toolbox CRP (40)]. Cross-recurrence quantification analyses were computed on the high-performance-computing center of Kiel University. The results are presented in mean ± standard deviation (SD). Significance levels were *p* < 0.05*, *p* < 0.01**, and *p* < 0.001***.

## Results

### Sample and Relationship Characteristics

At study initiation, mean age and mean relationship duration were 23.5 ± 3 years and 34 ± 28 months, respectively. Regular bed sharing had happened for a mean of 19.1 ± 11.7 months on 6.4 ± 1.1 nights per week prior to the study. Passionate love ratings reached 85 ± 8.9 of possible 105 points. Relationship quality, was rated at 3.7 ± 0.3 and 3.5 ± 0.3 for the support and depth dimension, respectively. The conflict dimension was rated 1.5 ± 0.3, indicating an overall low conflict level. The present sample scored significantly better on all QRI dimensions than the sample of the validation study of the German QRI (32) (all ps < 0.001; one sample, two-tailed t-tests against the means of the respective dimension ratings in the validation study).

Chronotype ratings (D-MEQ scores) ranged from 37 to 68 with a mean of 56 ± 7.8. There was no significant difference between males and females (*p* = 0.704). Within-couple differences in D-MEQ scores ranged from 0 to 31 (mean 7.9 ± 7.9; median 6.0). Seven couples had matching chronotypes, four differed by one category (either moderately morning type or moderately evening type vs. neither type), and one couple differed by two categories (moderate evening type vs. moderate morning type).

Detailed descriptive statistics of the sample are given in **Table 1**.

### Impact of a Partner's Presence on Classical Sleep Parameters and REM Sleep Fragmentation

#### Individual Sleep vs Co-Sleep

Detailed results comparing co-sleep vs. individual sleep are given in **Table 2**.

**Table 2:** Subjective and objective sleep parameters individual sleep vs. co-sleep.

	Mean		SD		*p* value
Subjective parameters	I	C	I	C	
Morning condition	13.4	13.2	2.1	2.7	0.524
Sleep onset latency [min]	20.2	18.1	14.4	13.3	*0.511*
Sleep time [min]	481.9	479.4	32.9	32.4	*0.485*
Number of awakenings [1/night]	2.9	2.6	1.7	1.1	0.224
**Polysomnography**				
Total sleep time [min]	467.6	467.1	27.0	20.6	*0.423*
Sleep efficiency [%]	92.0	92.3	5.1	3.2	*0.784*
Sleep onset latency [min]	10.6	11.8	7.6	11.3	*0.657*
REM sleep latency [min]	95.4	98.2	40.1	36.5	*0.852*
N1 sleep [% of sleep time]	8.4	7.7	3.6	2.2	0.325
N2 sleep [% of sleep time]	46.0	44.7	5.3	6.7	0.255
SWS [% of sleep time]	24.4	23.6	7.6	9.3	0.508
REM [% of sleep time]	21.0	23.0	4.2	4.2	**0.005**
Number of awakenings [1/night]	23.5	25.8	7.4	7.1	*0.148*
Isolated movements	50.9	61.5	32.9	36.7	***0.007***
Apnea Hypopnea Index (AHI)	1.3	1.3	1.8	1.9	*0.782*
Snoring events [1/night]	4.5	14.4	12.8	49.6	0.085

Individual sleep (I) and co-sleep (C) differed significantly with respect to %REM sleep and movements (bold p values). Given are mean and standard deviation (SD), tests employed were Wilcoxon signed-rank testes (p values in italics) or paired two-tailed Student's t-tests. Normal distribution was tested by the Shapiro-Wilk test (results not given).

There were no significant differences between co-sleep and individual sleep regarding total sleep time, sleep efficiency, and sleep onset latency (**Table 2**). Therefore, only fractions of each sleep stage of total sleep time (% of total sleep time) were further analyzed.

**Figure 1:**
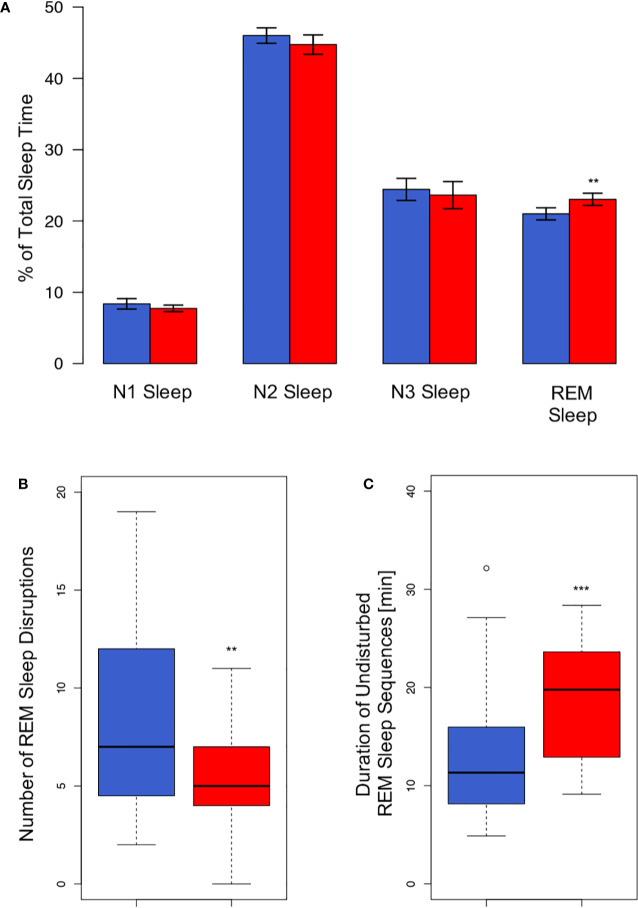
REM sleep alterations associated with the sleeping arrangement. **(A)** Co-sleep (red bars) is associated with an approximately 10% higher amount of relative REM duration (23 ± 0.9% vs. 21 ± 0.8%) as compared to sleeping alone (blue bars). No other sleep stage shows significant alterations associated with the sleeping arrangement. Given are means ± SEM. REM sleep is less fragmented under the co-sleep condition [red bar, panel **(B)**] which results in markedly longer undisturbed continuous REM sleep sequences **(C)**. Boxes represent first and third quartile (upper and lower margins) and median (bold horizontal line). N = 24, significance: ** < 0.01; *** < 0.001.

Under the co-sleep condition, couples showed significantly higher percentages of REM sleep as compared to sleeping alone (23.0 ± 4.2% vs. 21.0 ± 4.2%; *p*= 0.005, **Table 2**, **Figure 1A**). Moreover, REM sleep in presence of a partner was significantly less fragmented as compared to sleeping without a partner in the same room (5.4 ± 2.7 disruptions vs. 8.5 ± 5.2 disruptions; *p* = 0.008, **Figure 1B**). This translated into significantly longer undisturbed, continuous REM sleep fragments (22.0 ± 19.7 min vs. 13.4 ± 7.1 min; *p*= 0.0006, **Figure 1C**) during co-sleep. Also, co-sleeping was associated with a higher total number of leg movements (mean 61.5 ± 36.7) as compared to individual sleep (mean 50.9 ± 32.9; *p* = 0.007; **Table 2**). Controlling for multiple testing reduced *p* values of REM sleep percentage, number of REM sleep disruptions, continuous REM sleep fragments, and leg movements but did not lead to non-significant results of previous significant findings (*p* values after correction: 0.03, 0.03, 0.01, and 0.03, respectively).

In contrast, no significant difference was observed in any other sleep stage or any other monitored parameter besides REM sleep and movements (all ps > 0.148, for details see **Table 2**).

#### Relevance of Gender and Relationship Characteristics

**Figure 2:**
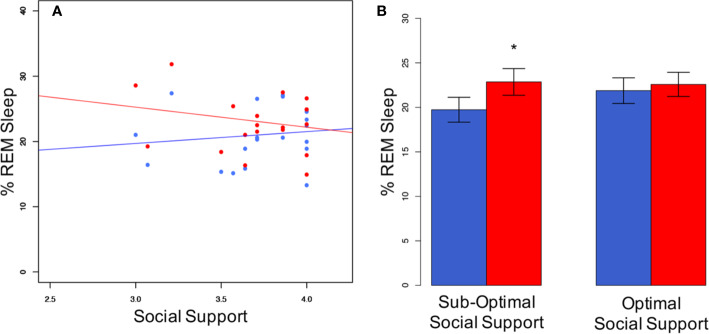
Social support interacts with sleeping arrangement regarding %REM sleep. **(A)** Individuals with not optimal social support levels show a greater difference in % REM sleep between co-sleep (red dots) and individual sleep (blue dots) than individuals with optimal social support. Pearson's correlations are non-significant for either of the both sleeping arrangements (individual sleep (blue line): *r* = 0.12; *p* = 0.567; co-sleep (red line): *r* = −0.21; *p* = 0.329). Note, that the individual with the lowest social support score (3.0) is still on the very supportive side. This translates into significant differences in the sub-optimal social support group in a median-split analysis of co-sleep **(B)**. N = 24, significance * < 0.05, given are mean ± SEM **(B)**.

Correlating REM-sleep percentage of the couples' individuals with each other (males ~ females) did not render significant results. This was true for both, individual sleep (*r* = −0.26; *p* = 0.419) and co-sleep (*r* = −0.36; *p* = 0.257). Both *p* values were higher than the “very liberal” (38) alpha of 0.25 which has been suggested as a reference in this type of calculation (38). Therefore, we concluded that the assumption of independence of AN(C)OVA can be justified. The consequently conducted AN(C)OVAs showed a significant interaction effect of sleeping arrangement and the “social support” subscale of the QRI regarding the percentage amount of REM sleep (F(1,22) = 4.8, *p* = 0.039, **Figure 2**)). No other relationship parameter (conflict, relationship depth, passionate love, relationship duration) interacted significantly with sleeping arrangement to explain the co-sleep-associated increase in REM sleep percentage of total sleep time (all Fs(1,22) ≤ 0.9, all ps ≥ 0.342). While the sleeping-arrangement variable represented a significant main effect throughout all calculations (all Fs(1,22) ≥ 9.0, all ps ≤ 0.007), none of the relationship characteristics did (all Fs(1,22) ≤ 0.3, all ps ≥ 0.567).

Similarly, the gender variable did not yield significant interaction or main effects (all Fs(1,22) ≤ 0.1, all ps ≥ 0.762).

### Synchronization

#### Synchrony at the Same Point in Time

**Figure 3:**
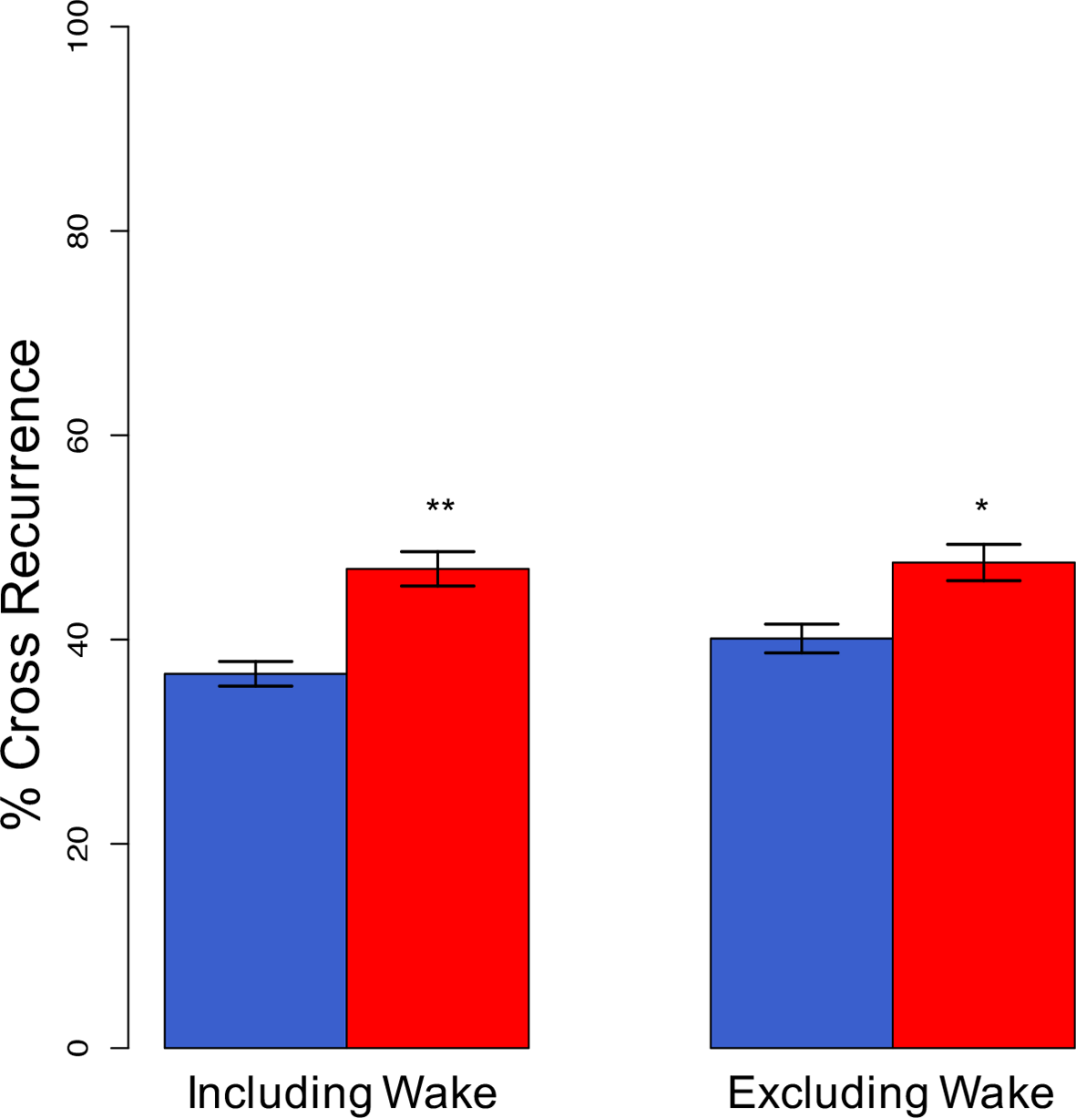
Synchronization of sleep stages at lag 0 (complete synchronization). Complete, direct synchronization of sleep stages is significantly increased in co-sleep (red bars) as compared to sleeping alone (blue bars) resulting in nearly half of the night's sleep being synchronized. The synchronization during co-sleep is independent of inclusion or exclusion of wake. N = 12, significance: * < 0.05; ** < 0.01, given are means ± SEM.

Coupling between partners was assessed using cross-recurrence quantification analysis (40). First, we analyzed sleep stage synchrony at the same point in time without considering lag and lead phenomena (**Figure 3**). Sleeping apart from each other was associated with 36.6 ± 6.0% of the night being synchronized. That increased significantly (*p* = 0.005) to 46.9 ± 8.4% when a partner was present. Excluding wake resulted in 40.1 ± 7.1% of epochs being synchronized during individual sleep and 47.5 ± 8.9% in co-sleep (*p* = 0.022).

#### Lead and Lag Phenomena

**Figure 4:**
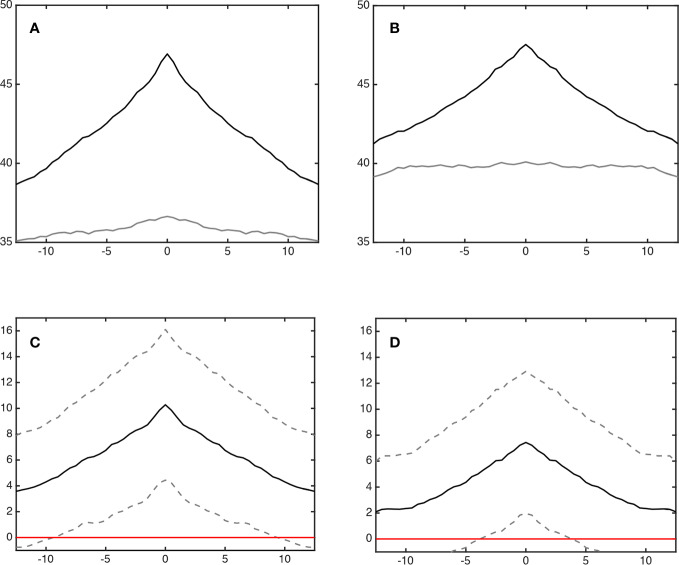
Coupling of sleep architecture (lag synchronization). Panels **(A**, **B)** show the synchronization (% cross recurrence, y-axis) during co-sleep (upper black line) and individual sleep (lower grey line) across lags (minutes, *x*-axis). Co-sleeping is associated with a symmetrical incline of synchronization across ± lags peaking at lag 0 at 46.9 ± 8.4% (**A**; including wake) and 47.5 ± 8.9% (**B**; excluding wake), respectively. Individual sleep excluding wake **(B)** shows no peak at all. Including wake, **(A)** a minimal peak at lag 0 can be observed - possibly due to wake before sleep onset. Panels **(C**, **D)** show the difference in synchronization (co-sleep – individual sleep, black line). Dashed lines represent 95% confidence intervals. Coupling during co-sleep is significantly increased as compared to sleeping alone starting approximately at lag ± 10 min when wake is included **(C)** and app. ± 5 min without considering wake **(D)** as indicated by crossing of the lower dashed line with the red zero line.

**Figure 4** shows the average degree of sleep stage coupling across lags for each sleeping arrangement. Regardless of whether or not wake was included in the analysis, co-sleeping was associated with an increase in sleep stage synchronization across lags, peaking at lag 0 (**Figures 4A, B**, black lines). During individual sleep, only a minimal peak at lag 0 could be observed if wake was included (**Figure 4A**, gray line). If wake was excluded any dynamics in coupling across lags was missing (**Figure 4B**, gray line). It seems likely that the minimal peak during individual sleep including wake was due to wake before sleep onset.

Regarding statistical significance of coupling across lags, **Figures 4C, D** show that the increase in coupling of sleep stages during co-sleep vs. individual sleep reached significance at approximately lag ±10 min (including wake) and lag ±5 min (excluding wake), respectively.

#### Relationship Characteristics, Chronotype Similarity, Leg Movements, and Snoring

**Figure 5:**
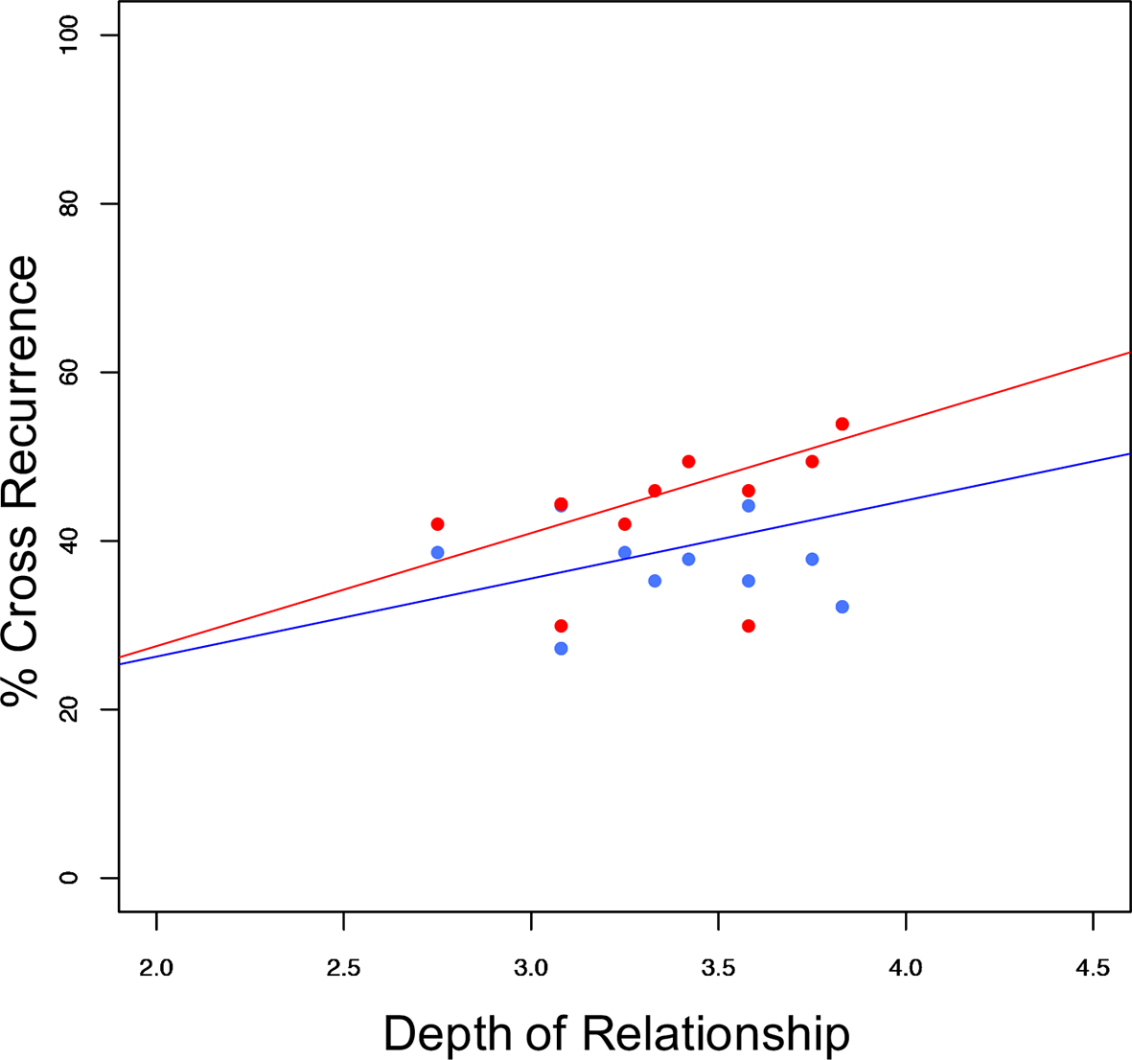
Sleep stage synchronization as a function of relationship depth. Distribution of synchronization (excluding wake) in relation to depth of relationship (couples' mean) and sleeping arrangement (co-sleep= red dots, individual sleep= blue dots) resulting in significant main effects of sleeping arrangement (F(1:10)= 6.585; *p* = 0.028), and relationship depth (F(1:10) = 5.976; *p* = 0.035) with no significant interaction (F(1:10) = 0.224; *p* = 0.646). Pearson's correlations of the respective sleeping arrangements are *r* = 0.45; *p* = 0.138 for individual sleep (blue line) and *r* = 0.52; p = 0.083 for co-sleep (red line) N = 12.

Finally, we investigated whether relationship characteristics, similar chronotypes, acoustic (snoring) or movement stimuli influence synchronization. With synchrony at lag 0 (excluding wake) as dependent variable, there was a significant main effect of the mean relationship depth between the partners (F(1,10) = 6.0, *p* = 0.035). The relationship between synchronization, depth of relationship, and sleeping arrangement is given in **Figure 5**. None of the other analyzed parameters (social support, conflict, passionate love, relationship duration, chronotype similarity, snoring, or leg movements) yielded significant main effects or interactions (all Fs (1,10) ≤ 2.8, all ps ≥ 0.127). In all investigated cases, sleeping arrangement represented a significant main effect (all Fs (1,10) ≥ 6.6, all ps ≤ 0.028).

## Discussion

The present work expands and complements the two previous studies that have polysomnographically investigated co-sleep vs. individual sleep of couples (17, 18). It includes relationship characteristics, chronotype, and gender in the analysis. Also, it clarifies contradictory findings of the previous works:

In a small pilot study, our group reported co-sleeping to be associated with a greater amount of REM sleep, SWS, total sleep time, a higher sleep efficiency, shorter N2 and N3 latencies as well as subjectively improved sleep quality (17). In contrast, Monroe's early sleep-laboratory-based study in 14 married good-sleeping young couples reported more moderate alterations (18). Except for a greater amount of REM sleep and awakenings and lower levels of S4 sleep during co-sleep no other subjective or objective sleep parameter was subject to partner-associated alterations. Also, there was no significant interaction of sleeping arrangement and gender regarding any sleep parameter (18). The present study supports Monroe's work to a large extent. This concerns parameters that are insusceptible to a changing sleeping arrangement, parameters that undergo partner-associated alterations, as well as a lacking interaction of sleeping arrangement and gender. (It is of note that S4 sleep was not assessed in the present study due to differing sleep stage classifications, and the negative findings of the interaction analyses need to be treated cautiously due to a small sample size).

Besides these differences between the previous works, there is one sleeping-arrangement-dependent alteration in objective sleep parameters that is present across both previous studies and the present work: a greater amount of REM sleep during co-sleep. Interestingly, this partner-effect on REM sleep doesn't seem to be limited to humans. It has recently been reported for the hyrax, a socially living mammal (41). The authors of that study propose a biophysical mechanism, namely a partner-driven stabilization of ambient temperature as being causative for the promotion of REM sleep (41). Our analyses suggest psychosocial factors, i.e., social support, to be relevant, too. Another potential mechanism to be considered in future studies is how a partner alters stress levels before and during sleep. Presence of a partner might facilitate perceiving a sleeping environment as “safe”, whereas sleep in isolation might represent a stressor. Psychosocial stress has been reported to fragment REM sleep and might promote insomnia (42). Moreover, it has been shown in rats that sociality improves stress resilience by stabilizing REM sleep. After receiving electric shocks for purpose of fear conditioning, socially isolated rats reacted to that stressor with fragmented REM sleep. In contrast, rats that were having contact to a partner showed increased and undisturbed REM sleep (43).

Beyond the significant overlaps between Monroe's and the present work there are few but noteworthy differences. First, unlike Monroe, we do not find a significant difference in awakenings between individual sleep and co-sleep. It is however of note, that co-sleepers do wake up more often in the present study and albeit not statistically significant (*p* = 0.15) a Cohen's d of 0.5 indicates a medium effect size. (The effect size was calculated in R using the *lsr* and *pwr* packages). Second, Monroe does not report limb movements which in the present study are significantly more frequent during co-sleep as compared to individual sleep. This finding is in line with actigraphic studies of co-sleeping couples (3) and illustrates the pitfalls of interpreting actigraphic data. The increase in actigraphic movements has led to the conclusion that bed-sharing disturbs sleep objectively [e.g., (44)]. The present study – together with Monroe's work—challenges that view: despite the increase of movements (and awakenings), sleep architecture, and sleep-stage physiology remain intact during co-sleep, and REM sleep is stabilized and promoted. Thus, the present work supports Monroe's conclusion that the presence or absence of a partner might induce alterations that are distinct from the usual correlates of good and bad sleep (18).

Regarding the implications of these findings, two seem particularly relevant. First, REM sleep is known to benefit memory formation particularly of emotionally salient (45, 46) and episodic memories (47) [for review see (9)]. The latter (48) or both (49) have been linked to sociality. Moreover, imaging studies show that REM sleep is associated with an activation of—among others—the amygdala and the medial prefrontal cortex, the latter of which is part of the theory-of-mind network and therefore highly important for social cognition (50). Therefore, REM sleep might increase our preparedness and fitness to navigate the social world. Connecting this hypothesis to the findings of our study leads us to propose the existence of a positive feedback loop of REM-sleep-sociality interactions: social sleep enhances and stabilizes REM sleep which in turn enhances our ability to interact socially.

The second implication concerns potential mental health effects of the here reported findings. Partnerships have been shown to protect from mental illness (1) and it has been argued that sleep might be a mediator of health effects of relationships (2). On a sleep stage level, REM sleep might be of particular interest in this context. REM sleep is related to dissolving emotional stress (51) and balancing fear-related amygdala reactiveness (52, 53). Moreover, REM sleep fragmentation is related to insomnia (42), which in turn is a risk factor for developing a mental illness [e.g., insomnia doubles the risk for depression (54)]. Therefore, REM-sleep stabilization due to co-sleep might mediate (or moderate) the established effect of partnerships on mental health.

Besides displaying neurophysiological changes (increased and stabilized REM sleep), sleeping in company is subject to interactive dyadic effects. Recently, the combination of dual simultaneous polysomnography and cross-recurrence quantification analysis has been established by our group in order to study sleep-stage synchronization of co-sleeping couples (17). The present study reports increased sleep-stage synchronization independent of wake between co-sleeping partners as compared to sleeping alone. That basic finding reproduces prior findings (17) and adds important insights to the understanding of co-sleep. First, unlike the previous study, the subjective and objective sleep data in the present work do not indicate a general improvement in sleep quality by co-sleeping. Therefore, it can be ruled out that the increase in synchrony is a mere byproduct of better (i.e., less disturbed) sleep. Second, we show that coupling of sleep stages is not only a matter of direct synchrony, but spans a ± 5 min interval around lag 0. Third and fourth, synchronization is positively related to perceived relationship depth and independent of chronotype similarity. While the present study is the first to report this for sleep-stage synchrony, and thus for neuronal synchronization during sleep the latter both findings have been reported for actigraphically measured sleep-wake patterning in couples (7). Moreover, the relevance of relationship characteristics links sleep-stage synchrony to neuronal synchronization during wakefulness which has been reported to be modulated by affection and attachment style (55). That seems to be of great interest as neural synchronization during wake is relevant to core processes of human sociality such as interactive teaching and learning (56), joint action (57, 58), prosociality (59), or emergence of leadership in groups (60). Moreover, interpersonal synchronization has been linked to increased prosocial behavior, perceived social bonding, social cognition, and positive affect [for review see (19)]. The latter both are frequently impaired in mental illnesses such as schizophrenia and depressive disorder. Therefore, our results call for further investigating the role of sleep-related synchronization in mental illness. Given that there is in fact an observable relation of synchronization during sleep and mental-illness parameters (e.g., symptom severity, social functioning), synchronization might—depending on the causal direction—either represent another mechanism through which co-sleeping with a partner prevents mental illness and its social consequences, or it might be a symptom of mental illness that could represent a link between individual sleep disturbances and social deficits. Additionally, our findings might stimulate new research into mechanisms that underlie synchronization during wake since eye-to-eye contact (55, 61) or shared intentionality (58) – that are major mechanism how individuals synchronize – are not present or largely reduced during sleep.

While the findings of the present work are important, and the present study has some strengths (e.g., the methodological setup including a well-controlled lab-setting, the sufficient statistical power for direct comparison of sleep parameters, and advanced statistical analyses allowing for the analysis of lead and lag phenomena, or the inclusion of relationship characteristics, chronotype, and gender) it is also limited to some respects that are mostly related to the explorative nature of some of the analyses.

The first limitation concerns the methodology. Laboratory-based polysomnography allows for high-quality and in-depth assessment of sleep. Yet, we can only speculate how our results relate to actigraphy which has been used by other works investigating couples' sleep in a more natural setting and over a longer period (3, 4, 62). A combination of in-lab polysomnography and 2 weeks of actigraphy would have allowed for integrating the actigraphic and polysomnographic literature on couples' sleep. A second limitation, that is related to the methodological setup, is the comparably small sample size for conducting two-way mixed ANOVAs and ANCOVAs. Thus, negative findings in the exploratory part of the present work need to be treated cautiously. Also, we did not adjust for multiple testing in the exploratory part in order to not reject possible effects prematurely. Therefore, future studies with an increased sample size should retest some of our findings regarding the effect of sound (snoring), movements, and chronotype similarity on interpersonal synchronization. This holds also true for relationship quality. Moreover, a wider range of relationship characteristics could be included in future works. Third, we did not analyze the data on a dyadic level. This was done to ensure comparability with the previous polysomnographic studies. Also, we tested and confirmed that this approach is adequate since independence of the data was not to be rejected after correlating the partners with each other. Nevertheless, a dyadic statistical approach [as e.g., presented by Kenny (63)] might render interesting insights into couple dynamics during sleep, and future studies with an increased sample size should consider this approach. Fourth, while there is good reason to believe that a more stable REM sleep would impact REM-sleep-dependent outcomes such as memory consolidation, dissolving of emotional stress or fear-related amygdala reactiveness – we did not test for such effects. The fifth limitation concerns the question of generalizability. We investigated young healthy heterosexual human couples in a lab-setting. Even though a social-sleep-related increase in REM sleep has been reported for other mammals (41), it is unclear whether a similar pattern of stabilized REM sleep, no other sleep stage alterations, increased movements or awakenings, and sleep-stage synchronization similarly occurs in other species, age groups, couples including one suffering from a disease, or in other social sleep constellations such as homosexual couples. Also, it is unclear whether the findings would also be present in a non-lab-setting i.e., at home. It is, however, of note that there are also good reasons to believe that the effect would be more pronounced in the usual home environment, e.g., the use of two-duvets in the present study or a presumably less intimate behavior in the lab.

In conclusion, despite some limitations the presented study reports novel findings regarding co-sleep-associated changes in sleep architecture and synchronization. Social support and relationship depth might be important co-factors. Thereby, the present study raises important questions to be elucidated in the future, namely, whether the co-sleeping-induced REM sleep stabilization is i) part of an evolutionary important positive feedback loop of sleep and sociality, and (ii) alongside with interpersonal synchronization—a mechanism through which relationships prevent mental illness.

## Data Availability Statement

The datasets generated for this study are available on request to the corresponding author.

## Ethics Statement

The studies involving human participants were reviewed and approved by Ethik-Kommission der Medizinischen Fakultät der Christian-Albrechts-Universität zu Kiel. The participants (on patients were included) provided their written informed consent to participate in this study.

## Author Contributions

Conceptualization: HD, PM, SW, AR, RG. Design and methodology: HD, PM, AR, RG, SW, SLW, PCB, HB-J, PB. Conduction of the study: PB, HB-J, HD. Statistical analysis and interpretation: HD, SW. Writing—original draft preparation: HD. Writing—review and editing: SW, PB, HB-J, SLW, PM, PCB, JL, RG. Resources: RG. Supervision: RG, AR. All authors contributed to the article and approved the submitted version.

## Funding

The study was funded by own resources of the Department of Psychiatry and Psychotherapy, Christian-Albrechts-University Kiel, Kiel, Schleswig-Holstein, Germany. Open access publishing was supported by the German Research Foundation (DFG) within the funding programme "Open Access Publizieren".

## Conflict of Interest

The authors declare that the research was conducted in the absence of any commercial or financial relationships that could be construed as a potential conflict of interest.
